# Serum dickkopf-1 is a novel serological biomarker for the diagnosis and prognosis of pancreatic cancer

**DOI:** 10.18632/oncotarget.4529

**Published:** 2015-06-19

**Authors:** Su-xia Han, Xia Zhou, Xin Sui, Chen-chen He, Meng-jiao Cai, Jin-lu Ma, Yuan-yuan Zhang, Cong-ya Zhou, Chen-xian Ma, Armando Varela-Ramirez, Qing Zhu

**Affiliations:** ^1^ Department of Oncology, The First Affiliated Hospital of Xi'an Jiaotong University Medical College, Xi'an, Shaanxi, PR China; ^2^ Department of Biotherapy, The Fourth Affiliated Hospital of China Medical University, Shenyang, Liaoning, PR China; ^3^ Department of Biological Sciences and Border Biomedical Research Center, The University of Texas at El Paso, El Paso, Texas, USA

**Keywords:** pancreatic cancer (PC), dickkopf-1 (DKK1), serum biomarker, diagnosis, prognosis

## Abstract

**Purpose:**

To identify whether Dickkopf-1 (DKK1) could be a potential biomarker for early detection and prognosis in patients with pancreatic cancer (PC).

**Methods:**

Serum was collected from 140 patients with pancreatic adenocarcinoma and 92 control patients without pancreatic adenocarcinoma. Serological levels of DKK1 were examined by enzyme-linked immunosorbent assay (ELISA). The sensitivity and specificity was compared with carbohydrate antigen 19-9 (CA19-9). A 2-year follow-up was monitored to evaluate the correlation between DKK1 serum levels and overall survival. The expression of DKK1 in PC tumor tissues was also evaluated using immunohistochemistry staining.

**Results:**

Serum levels of DKK1 and CA19-9 were elevated in PC patients in the early-stage cases. These levels increased with the advancement of clinical stage. There was significant difference in DKK1 serum levels between early and advanced PC stages. Receiver operating characteristic curve (ROCC) analysis showed that DKK1 was significantly better than CA19-9 in differentiating patients with PC from the controls (area under the curve (AUC) 0.919 *versus* 0.853, respectively), especially in distinguishing early-stage cancer from chronic pancreatitis (CP). The expression of DKK1 in PC tissues correlated with its expression in serum samples. The overall survival rate was 24.4% in the group with higher DKK1 levels and was found to be significantly different from the group with lower DKK1 levels (33.3%).

**Conclusion:**

DKK1 may be a novel diagnostic/prognostic biomarker for PC.

## INTRODUCTION

Pancreatic cancer (PC) is one of the top-five leading causes of cancer-related death and has the lowest survival rate among the solid tumor cancers. PC accounts for an estimated 38,460 deaths per year worldwide [1, 2]. In spite of the scientific advances of modern surgical techniques, only about 4% of patients with PC will live 5 years after the initial diagnosis [3]. The poor survival rate is attributed to the fact that PC is often diagnosed in advanced phases of the disease. Only 10% of PC cases are considered suitable for surgical resection, which at present offers the only chance of survival. Unfortunately 90% of patients present with unresectable stage III/IV at the time of diagnosis and most had a median survival time of less than 1 year post-diagnosis [3, 4]. Currently, one of the most widely used serum markers for the diagnosis of PC is carbohydrate antigen 19-9 (CA 19-9). CA19-9 is highly inaccurate as a diagnostic test and it also fails to identify patients with small resectable tumors. The poor prognosis and high mortality in pancreatic cancer is in part due to delays in diagnosis as early symptoms are non-specific and most patients are not diagnosed until the disease is in an advanced stage. Therefore, the identification of novel biomarkers that can help detect PC at the earlier stages of the disease is urgently needed. Such findings could improve the prognosis and survival of PC patients.

Dickkopf-1 (DKK1) is a soluble inhibitor of Wnt/β-catenin signaling that is required for embryonic head formation in vertebrate development [5-7]. DKK1 regulates Wnt signaling pathway by binding to lipoprotein-related protein-5/6 (LRP5/6) and Kremen proteins and induces LRP endocytosis, which prevents the formation of Wnt-Frizzled-LRP5/6 receptor complexes. This allows entry of β-catenin into the nucleus where it interacts with members of T cell factor (TCF) family, which regulate Wnt target genes that are essential for embryonic development and tumorigenesis. Mutations that promote constitutive activation of the Wnt signaling pathway lead to cancer. A number of studies have reported over-expression of DKK1 in multiple myeloma, hepatocellular carcinoma, prostate cancer, breast cancer, gastric cancer, and lung cancer [8-15]. The inhibition of Wnt signaling by DKK1 is a frequent event in diverse human cancer. We propose that serum DKK1 levels may serve as potential diagnostic and prognostic biomarkers in patients affected with PC. The aim of this study was to examine the diagnostic value of DKK1, correlating its serological levels with tumor stage and survival rate in PC patients.

## RESULTS

### Participants characteristics

A total of 140 PC patients and 92 controls were eligible for this study. The median age for the participants was 68 years; 65.5% were men. Demographic characteristics were well balanced between the two groups (Table 1). This study included 16, 46, 27, and 51 PC patients with stage I, II, III, and IV, respectively. In addition, 62 (44.3%) of the PC patients were in early-stage of the disease. There were 92 non-pancreatic cancer individuals consisted of 48 healthy subjects, 18 benign pancreatic tumors (BPT) patients, and 26 chronic pancreatitis (CP) patients.

**Table 1:** Clinicopathological characteristics of recruited participants (n=232)

	Pancreatic Cancer	Control			
		Healthy Control	Benign pancreatic tumor	Chronic Pancreatitis	*P*
Variable	(n=140)	(n=48)	(n=18)	(n=26)	
Age, year					
Mean±SD	61.7±10.8	59.1±11.0	58.8±12.1	57.6±11.3	> 0.05
Range	36-84	34-79	38-78	40-79	
Sex, n(%)					
Male	88 (62.9%)	30 (62.5%)	12 (66.7%)	22 (84.6%)	> 0.05
Female	52 (27.1%)	18 (37.5%)	6 (33.3%)	4 (15.4%)	
ALT, n(%)					
≤ 40	61 (43.6%)	46 (95.8%)	16 (88.9%)	20 (76.9%)	> 0.05
＞40	79 (56.4%)	2 (4.2%)	2 (11.1%)	6 (23.1%)	
GTT, n(%)					
≤ 40	65 (46.4%)	45 (93.8%)	15 (83.3%)	20 (76.9%)	> 0.05
＞40	75 (55.6%)	3 (6.2%)	3 (16.7%)	6 (23.1%)	
Tumor size, n(%)					
≤2.0 cm	11 (7.8%)				
2.0-5.0 cm	45 (32.1%)				
5.0-8.0 cm	25 (17.8%)				
＞8.0 cm	13 (9.3%)				
Missing	46 (33.0%)				
TNM stage(AJCC)					
I	16 (11.4%)				
II	46 (32.9%)				
III	27 (19.2%)				
IV	51 (36.5%)				
Tumor Differentiation					
I/II	55 (39.3%)				
III/IV	33 (23.6%)				
Missing	52 (37.1%)				

ALT, alanine aminotransferase; GGT, gamma-glutamyl transpeptidase;

TNM, T, Tumor; N, Node ; M, Metastasis; AJCC, American Joint Committee on Cancer Staging;

### Serum level of CA19-9 and DKK1 in PC patients and control group

**Figure 1:**
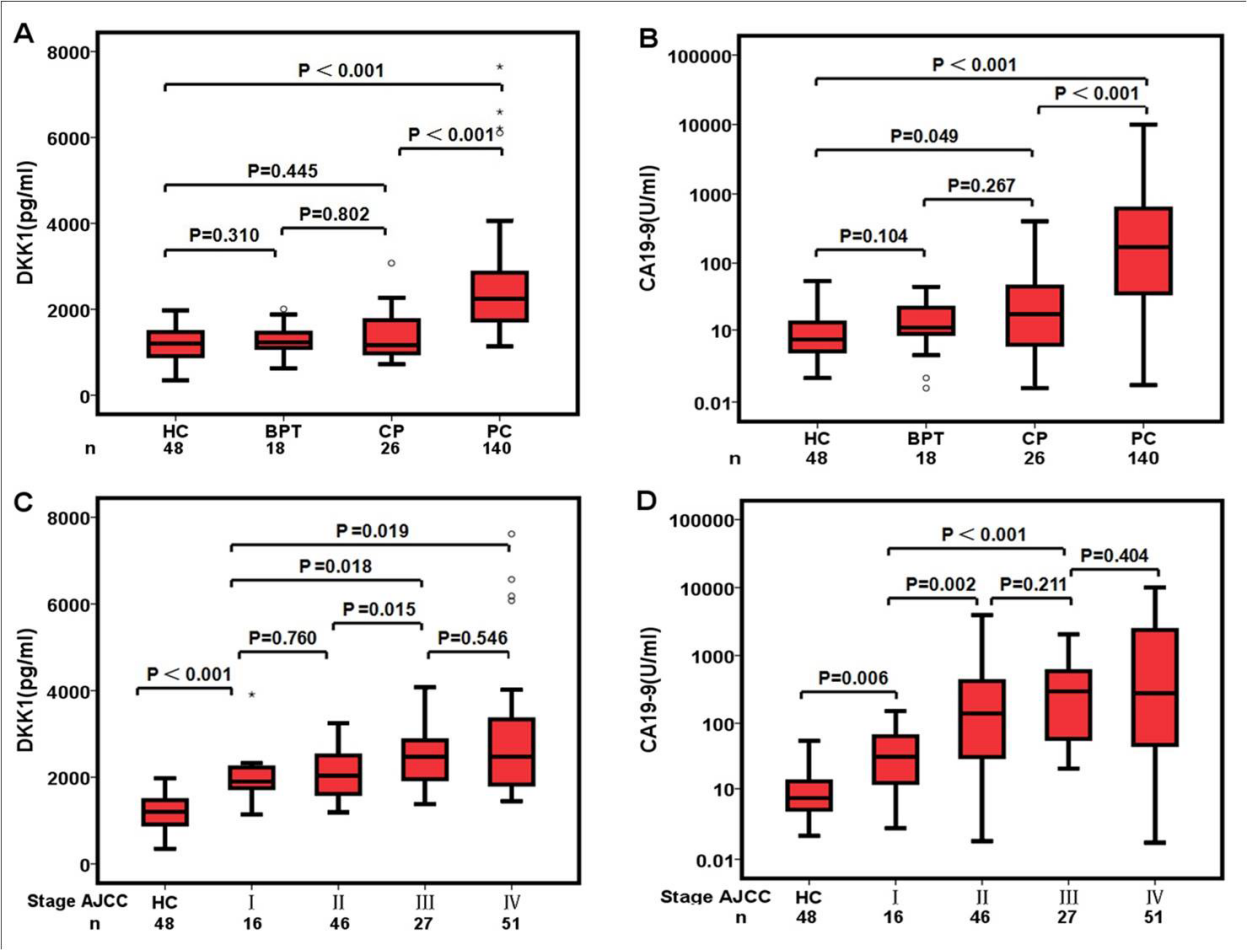
Pre-operative serum concentrations of DKK1 and CA19-9 **A.** Serum DKK1 and **B.** serum CA19-9 levels in various pancreatic diseases. **C.** Serum DKK1 and **D.** Serum CA19-9 levels in various stage of pancreatic cancer. A logarithmic function (log10) was used for the CA19-9 (U/ml) Y-axis.

Serum levels of CA19-9 and DKK1 in PC patients and control groups were shown in Table 2. Compared with the control groups, the serum level of CA19-9 (median, range) was significantly higher in the cancer group (170 pg/ml, 0.87-10000 *vs* 15 pg/ml, 0.60-404, *p* < 0.05). Serum DKK1 protein levels were also significantly higher in PC patients as compared to controls (2243 pg/ml, 1136-7653 *vs*. 1212 pg/ml, 348-3090, *p* < 0.05, Figure 1). There were no significant differences in serum concentrations of CA19-9 and DKK1 between HC, BPT, and CP groups. Greater levels of both DKK1 and CA19-9 were observed in stage I of PC patients compared to the HC group (Figure 1). And DKK1 levels were increased with the advancement of clinical stages. There were significant differences in DKK1 between early PC stage and advanced PC stage. Differences in DKK1 serum levels between CP and stage I PC (1165 pg/ml, 719-3090 *vs* 1901 pg/ml, 1136-3945) also achieved statistical significance (*p* < 0.001). There was no statistical differences in CA19-9 levels between groups (Figure 1).

**Table 2:** Serum DKK1 and CA19-9 levels in 232 study participants

Characteristics	DKK1(pg/ml)	CA19-9(U/ml)
Healthy Controls(*N* = 48)		
Mean±SD	1182.52±351.12	14.03±15.85
Median	1201.46	7.02
Range	348.54-1974.24	1.23-54.60
Benign pancreatic tumors(*N* = 18)		
Mean±SD	1293.99±363.08	15.02±11.52
Median	1229.46	10.94
Range	625.42-2006.85	0.60-45.05
Chronic pancreatitis(*N* = 26)		
Mean±SD	1361.61±574.66	40.03±78.26
Median	1165.24	17.67
Range	719.00-3090.46	0.60-404.10
Pancreatic cancer(*N* = 140)		
Mean±SD	2445.09±1.01E3	947.65±1998.69
Median	2243.04	170.56
Range	1136.58-7653.98	0.87-10000.00

Note: DKK1, Dickkopf-1; CA19-9, Carbohydrate antigen

### Diagnostic value of serum CA19-9 and DKK1

The 140 PC patients were categorized according to AJCC Stages and compared serum levels of CA19-9 and DKK1 in each stage (Table 3). Our results showed that serum CA19-9 levels in PC patients with advanced stage III/IV were markedly elevated. And significant difference was showed between serum CA19-9 levels in PC patients with stages I/II (*n* = 16/46) and healthy controls (*P* < 0.001). Furthermore, there was the significant difference between serum DKK1 levels in patients with early-stage pancreatic cancer (stages I/II) and those in healthy controls (*P* < 0.001). In addition, eight patients with stage I pancreatic cancers showed elevated DKK1 levels in conjunction with normal CA19-9 serum levels (Data not shown).

**Table 3:** Serum DKK1 and CA19-9 levels in each stage of 140 pancreatic cancer patients

AJCC stage	DKK1(pg/ml)	CA19-9(U/ml)
I A/B (*N* = 16)		
Mean±SD	2052.31±595.33	44.23±43.56
Median	1901.36	31.72
Range	1136.58-3945.65	1.89-151.60
II A/B (*N* = 46)		
Mean±SD	2088.94±579.21	526.73±945.90
Median	2035.11	139.8
Range	1189.93-3248.10	0.87-3943.00
III (*N* = 27)		
Mean±SD	2501.35±702.31	447.69±538.68
Median	2473.20	296.90
Range	1376.76-4075.04	22.10-2046.00
IV (*N* = 51)		
Mean±SD	2859.76±1352.87	1875.40±2951.54
Median	2475.85	278.40
Range	1446.61-7653.98	0.97-10000.00
Total(*N* = 140)		
Mean±SD	2445.09±1.01E3	947.65±1998.69
Median	2243.04	170.56
Range	1136.58-7653.98	0.87-10000.00

AJCC, American Joint Committee on Cancer Staging; SD, Standard deviation

**Figure 2:**
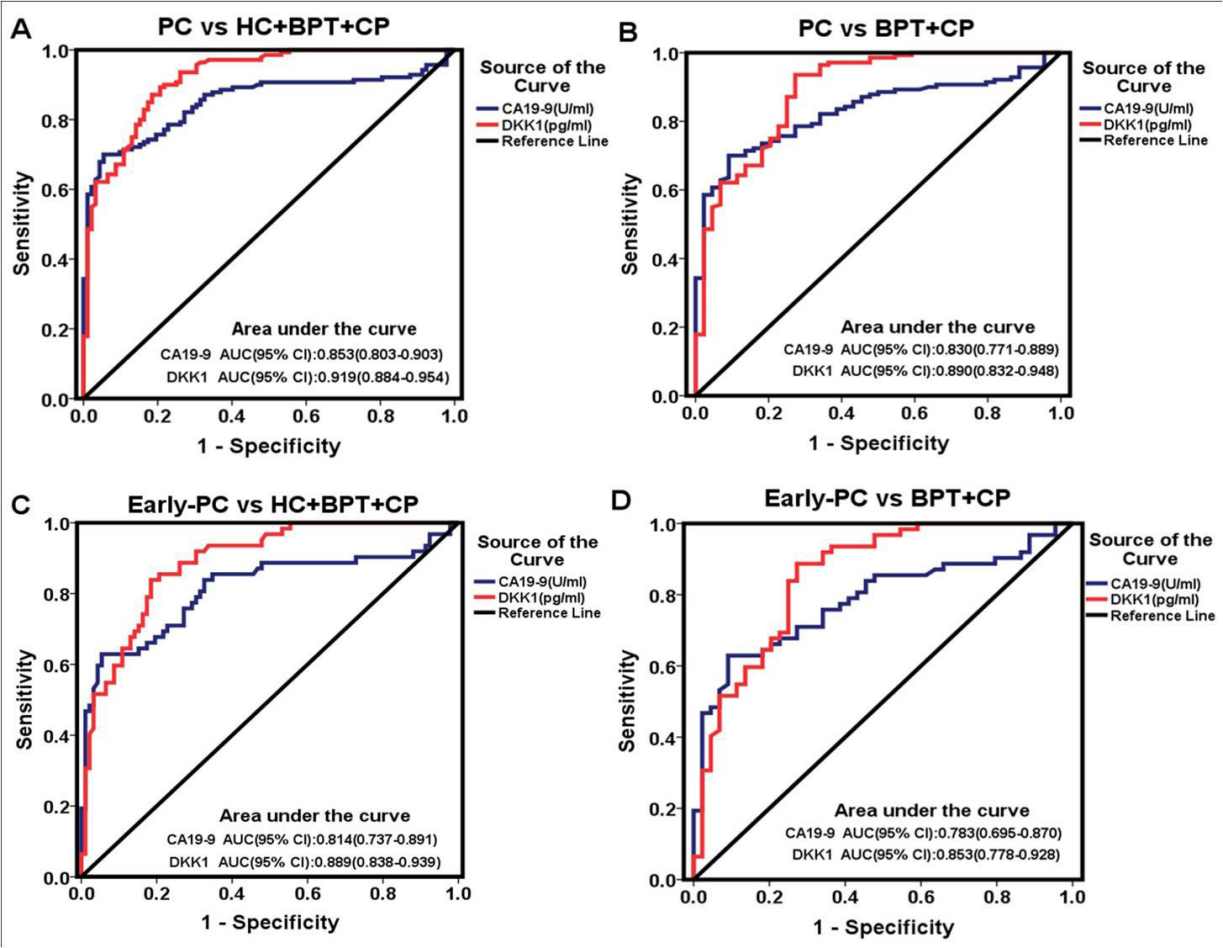
Diagnostic outcomes of pre-operative serological levels of DKK1 (red line) and CA19-9 (blue line) in the diagnosis of PC and early-PC **A.** ROC curve for DKK1 and CA19-9 for patients with PC versus all controls; **B.** ROC curve for DKK1 and CA19-9 for patients with PC versus BPT and CP; **C.** ROC curve for DKK1 and CA19-9 for patients with early-PC versus all controls; **D.** ROC curve for DKK1 and CA19-9 for patients with early-PC versus BPT and CP.

The diagnostic value of serum DKK1 for PC was evaluated by ROC curves analysis. Sensitivity, specificity, and all cutoff values of CA19-9 and DKK1 levels were determined using ROC analysis. Comparing PC patients with healthy controls, the best cutoff level of CA19-9 and DKK1 was 39.3U/ml and 1560.02pg/ml. So the cutoff of 1560 pg/ml was selected to categorize patients as higher or lower serum DKK1 level. Results for measurement of serum DKK1, CA19-9, or both were showed in the diagnosis of PC (Table 4, Figure 2). The accuracy of DKK1 serum levels (85.3%; sensitivity 89.3%, specificity 79.3%) was higher than CA19-9 (77.6%; sensitivity 73.6%, specificity 83.7%) and the AUC for DKK1 (0.919, 95% CI: 0.884-0.954) was greater than CA19-9 (0.853, 95% CI: 0.803-0.903). After excluding HC, the AUC for DKK1 (0.890, 95% CI 0.832-0.948) was also greater than CA19-9 (0.830, 95% CI: 0.771-0.889). For early-stage PC, the AUC for serum DKK1 was greater than that of CA19-9 regardless of HC inclusion or exclusion. In addition, the sensitivity, accuracy and Youden's index for serum DKK1 were also better than those for CA19-9 (Table 4).

**Table 4:** Results for measurement of serum DKK1, CA19-9, or both,* in the diagnosis of PC and early phase PC (*N* = 232)

	AUC (95%CI)	Sensitivity (%)	Specificity (%)	Accuracy (%)	Youden's index	Positive LR	Positive LR
PC *vs* HC+BPT+CP							
DKK1	0.919(0.884-0.954)	89.29%	79.35%	85.34%	0.68	4.31	0.13
CA19-9	0.853(0.803-0.903)	73.57%	83.70%	77.60%	0.57	4.51	0.32
DKK1+CA19-9		96.43%	64.13%	83.62%	0.61	2.69	0.06
PC *vs* BPT+CP							
DKK1	0.890(0.771-0.889)	89.29%	72.73%	85.33%	0.61	3.26	0.15
CA19-9	0.830(0.832-0.948)	73.57%	81.81%	75.54%	0.54	4.04	0.32
DKK1+CA19-9		99.29%	56.18%	89.13%	0.55	2.27	0.01
Early-PC *vs* HC+BPT+CP							
DKK1	0.889(0.838-0.939)	85.48%	79.34%	81.82%	0.64	4.14	0.18
CA19-9	0.814(0.737-0.891)	64.52%	83.70%	75.97%	0.48	3.96	0.42
DKK1+CA19-9		98.39%	64.13%	77.92%	0.62	2.74	0.04
Early-PC *vs* BPT+CP							
DKK1	0.853(0.778-0.928)	85.48%	72.72%	80.19%	0.58	3.13	0.20
CA19-9	0.783(0.695-0.870)	64.51%	81.81%	71.70%	0.46	3.55	0.43
DKK1+CA19-9		98.39%	56.81%	81.13%	0.55	2.28	0.03

DKK1, dickkopf-1; CA19-9, Carbohydrate antigen; AUC, area under curve; LR, likelihood ratio; PC, Pancreatic cancer; HC, healthy controls; BPT, Benign pancreatic tumors; CP, chronic pancreatitis.

* The diagnostic cutoff values of serum DKK1 and CA19-9 were 1560.02pg/ml and 39.3/ml, respectively.

**Figure 3:**
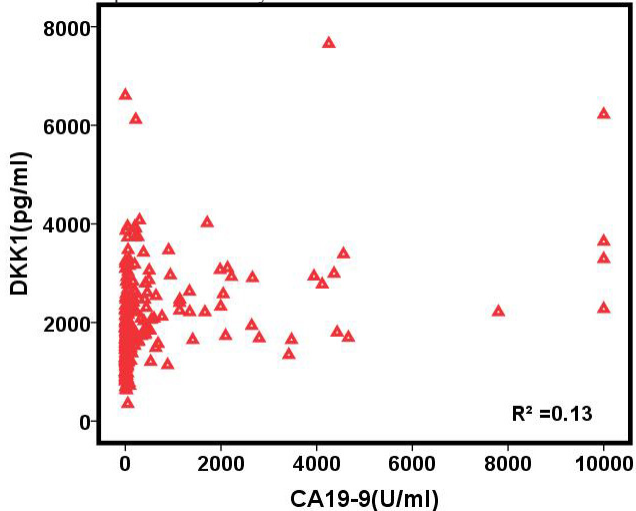
Scatter plot for pre-operative serum concentration of CA19-9 (X axis) and DKK1 (Y axis) There was no correlation between the serological levels of DKK1 and CA19-9 (correlation coefficient, R² = 0.13).

To determine how the serum biomarkers behaved in each group of subjects, we made scatter plots of serum CA19-9 and DKK1 levels (Figure 3). These data showed no correlation between serum DKK1 and CA19-9 levels, with a correlation coefficient (R^2^) of 0.13. Most significantly, this study suggested that serum DKK1 could possibly behave as a serum biomarker for PC patients.

### Serum DKK1 concentration before and after surgery

**Figure 4:**
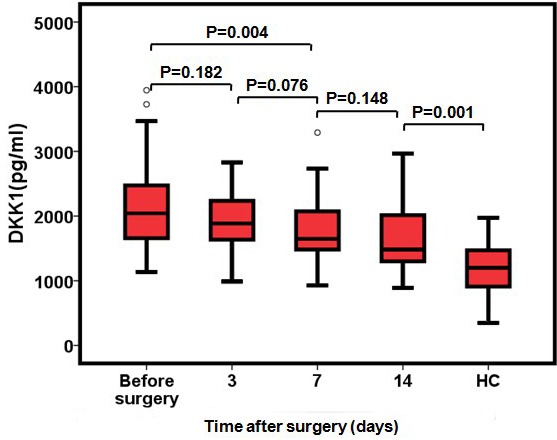
Serum concentrations of DKK1 after surgical resection of PC (*N* = 65) Serum DKK1 levels in the PC patients before surgery and 3, 7 and 14 days after surgery. DKK1 values from healthy controls are also included.

For 65 PC patients that underwent surgery with curative intent, serum DKK1 level decreased from 2151.67 pg/ml to 1936.57pg/ml (*p* = 0.18) at 3 days after surgery. Serum DKK1 levels achieved statistical significance at 7 and 14 days after surgery, compared with baseline (*p* < 0.05). Moreover, the level of DKK1 protein remained significantly higher than that of the HC group through 14 days (*p* < 0.001, Figure 4).

### Prognostic value of DKK1 levels before surgical resection for PC patients

**Figure 5:**
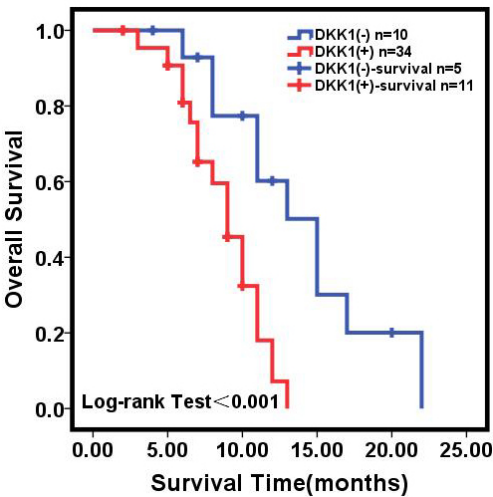
Kaplan-Meier analysis of overall survival of PC patients Kaplan-Meier analysis of overall survival (cumulative survival) of PC patients (*N* = 60) with surgical resection relative to pre-operative serum DKK1 concentrations.

A selected number of PC patients that underwent surgery (60 of 65) were eligible for survival analysis. Forty-four (44) of these patients were found to be deceased at the time of their 2-year follow-up. The overall median survival time after surgery was 9 months for the group with higher DKK1 levels and 15 months for the group with lower DKK1 levels (Log-Rank = 12.951, *p* < 0.001, Figure 5). The overall survival rate in the group with higher DKK1 levels was found to be 24.4%. This was significantly different from 33.3% survival rate in the group with lower DKK1 levels (Log-Rank = 12.951, *p* < 0.001, Figure 5).

### The relationship between the expression of DKK1 in PC tumor tissue and serum

**Figure 6:**
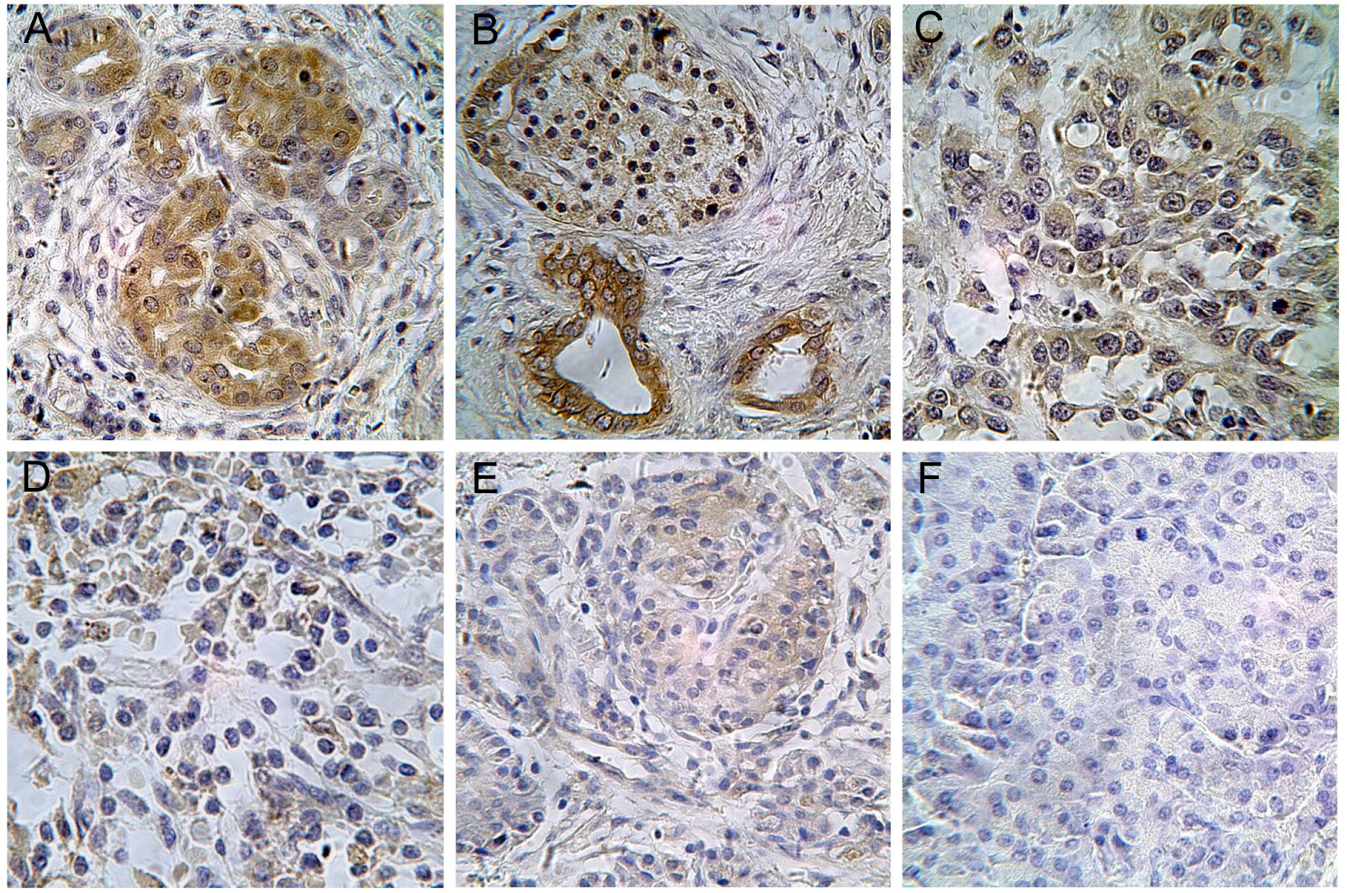
Immunohistochemistry (IHC) stained tissues of DKK1 in pancreatic diseases Tissues **A.** DKK1 expression in Well-differentiated ductal adenocarcinoma (stage II A) in a patient with serum DKK1 level of 2903.122pg/ml exhibited strong positive staining for DKK1 IHC; **B.** DKK1 expression in well-differentiated adenosquamous carcinoma (stage IIA) in a patient with serum DKK1 level of 3194.425pg/ml exhibited strong positive staining for DKK1 IHC; **C.** DKK1 expression in poor-differentiated ductal adenocarcinoma (stage IIB) in a patient with serum DKK1 level of 1613.621 pg/ml exhibited strong positive staining for DKK1 IHC; **D.** DKK1 expression in well-differentiated ductal adenocarcinoma (stage IIB) in a patient with serum DKK1 level of 1233.281pg/ml exhibited weak positive staining for DKK1 IHC; **E.** DKK1 expression in solid pseudo papillary tumor (SPT) in a patient with serum level of 1876.533 pg/ml DKK1 level exhibited weak positive staining for DKK1 IHC; **F.** DKK1 expression in chronic pancreatitis (CP) in a patient with normal serum DKK1 level exhibited no staining for DKK1 IHC. (Magnification x400).

To determine the prevalence and clinical significance of DKK1 in PC, we investigated DKK1 expression in 44 pancreatic specimens (included 36 PC and 8 other benign disease) by immunohistochemistry (Figure 6). The cutoff of 1560 pg/ml was selected to categorize patients as higher or lower serum DKK1 level. For the subjects with higher serum DKK1 level, 81.8% of cases had strong DKK1 staining in tissue; whereas only 9.1% cases with relatively lower serum DKK1 had strong DKK1 staining in tissue. Spearman rank correlation coefficients showed that serum DKK1 concentrations were positively associated with the expression of DKK1 in tissues (r = 0.713; *p* < 0.001, Table 5).

**Table 5:** The correlation between serum DKK1 level and DKK1 expression in the tissue specimens of the same PC patients

	DKK1 expression by IHC (N = 44)		
	strong positive	weak positive	negative
High serum DKK1	18	2	2
Low serum DKK1	2	4	16

## DISCUSSION

Patients afflicted with pancreas adenocarcinoma have an unfavorable survival rate with less than 4% living beyond 5 years from the intitial diagnosis [3]. In this study, we examined the potential utility of serum DKK1 levels as a diagnostic and prognostic marker for PC. Our results showed there is a higher DKK1 serological level in PC patients, especially in early stage, compared to CA19-9. DKK1 detection was also more efficient in diagnosis of early PC than CA19-9. Furthermore, the overall survival rate and median survival time was found to be notably shorter in the higher DKK1 group.

Early detection of PC remains a difficult diagnostic problem. The traditional PC biomarker, CA19-9, is inefficient for the diagnosis of early PC [17-19]. Other biomarkers were also reported with higher specificity but lower sensitivity as compared with CA19-9 [20, 21]. Recent emerging evidence indicates that using a panel of recombinant tumor-associated antigens (TAAs) could enhance the sensitivity and specificity of autoantibody detection in cancer [22, 23]. We (J Li et al, 2012) and others (R E Brand et al, 2011) also indicated that TAAs may be supplementary serological markers for the diagnosis of pancreatic cancer [24, 25]. However, the utility of a panel of autoantibody to TAAs in clinical setting is currently in its infancy, and the approach of anti-TAA arrays need to define systematically the optimal combination of TAAs. Clearly, a useful serum biomarker for PC is urgently needed.

DKK1 acts as a negative regulator of Wnt signaling. It can induce lipoprotein receptor-related protein (LRP) endocytosis, preventing the formation of the Wnt-Frizzled-LRP5/6 receptor complexes which leads to destabilization of ß-catenin and its subsequent degradation [26-29]. Over-expression of DKK1 is found in most human cancers, suggesting that it could serve as a useful diagnostic predictor for PC [14, 30-32]. Currently, there are no reports about the clinical significance of DKK1 protein as a serologic marker in PC. Our results showed that most PC patients displayed elevated serological levels of DKK1, even in those with early-stage PC. The diagnostic efficiency of DKK1 was better than CA19-9 as demonstrated in ROC curve analysis. This suggests that DKK1 is equivalent or even superior to the conventional diagnostic PC biomarker, CA19-9. The mechanism underlying DKK1 up-regulation in PC patients is unclear but there is some evidence indicating it might involve negative feedback of Wnt/β-catenin activation. In contrast to other studies, we found that DKK1 can also distinguish PC from CP. The availability of a noninvasive serum marker with satisfactory early diagnostic performance, like DKK1, could improve the clinical management and long-term outcome of PC.

Despite the limited number of PC patients involved in this study, we were able to determine that higher serum DKK1 levels before surgery correlated with less overall survival rate and overall median survival time. Takumi Yamabuki et al, reported similar performance of DKK1 in lung and esophageal cancer, and indicated that over-expression of DKK1 enhanced the cellular migration/invasive activity of cancer cell [32]. Thus, another explanation for the positive relationship between serum DKK1 levels and poor PC prognosis is that elevated DKK1 could enhance the metastatic potential of cancer cells.

We acknowledge there are inherent limitations in the nature of this sampling design, including time of follow up and patient sample size. Despite these limitations, there are a number of factors supporting our conclusions. The largest number of participants in this study was in the PC patient group which enabled correlative analysis of DKK1 expression in PC tumor tissues and serological DKK1 levels. In conclusion, serological levels of DKK1 could be used as a sensitive and non-invasive test for early detection and prognosis of PC. This assay may improve the existing conventional serodiagnostic assay in PC. However, further studies with long-term follow-up are needed to elucidate the diagnostic and prognostic value of serum DKK1 in PC patients. Additionally, large prospective investigations are required to provide more precise estimates of DKK1 sensitivity and specificity in PC.

## MATERIALS AND METHODS

### Patients population and setting

The study on PC was conducted between January 2010 and October 2013 in The First Affiliated Hospital of Xi'an Jiaotong University and The Tumor Hospital of Shaanxi province (Xi'an, P. R. China). All PC patients were histologically confirmed and were diagnosed according to the American Joint Committee on Cancer (AJCC). The clinical stage was judged using the International Union Against Cancer TNM classification. Patients were excluded if they had undergone radiotherapy or chemotherapy; or had a previous history of other cancer; or were suffering acute pancreatitis when this study was initiated. Furthermore, 65 PC patients after surgical resection were followed up over a two years period until all patients were deceased (At the end of the follow-up period 5 PC patients were lost to follow up). Ninety-two (92) patients without pancreatic adenocarcinoma served as controls and were divided into three groups including healthy control (HC; *n* = 48), patients with benign pancreatic tumors (BPT; *n* = 18), and patients with chronic pancreatitis (CP; *n* = 26). This study was approved by the Ethics Committee of the First Affiliated Hospital of Xi'an Jiaotong University. Written informed consent was obtained from all subjects.

### Study design and samples collection

All tissue samples of participants (PC patients, *n* = 140; controls, *n* = 92) were identified by two pathologists blinded to the diagnosis. From each participant, 5 ml of fasting blood were collected into tubes and transported immediately to the laboratory where it was stored at 4°C. Serum samples were obtained by centrifugation for 20 min at 3000 rpm and then divided into 3 to 5 aliquots and stored at −80°C until analyses. DKK1 level in serum from patients undergoing a surgical therapy was investigated before surgery, and 3, 7, 14 days after surgical resection. Primary PC tumor tissues were obtained from patients operated on with curative intent, fixed in 10% formalin, and embedded in paraffin. A follow-up was implemented to evaluate the patients' survival 2 years post-surgery.

### Enzyme-linked immunosorbent assay (ELISA)

Serum levels of DKK1 and CA19-9 were measured by enzyme-linked immunosorbent assay (ELISA) kits (R&D Systems, Minneapolis, MN). Experiments were set up and performed according to manufacturers' instructions. Absorbance values were read at a wavelength of 450nm using 96-well microplate reader (Thermo Systems, Boston, MA, USA). Each experimental point was performed in triplicate.

### Immunohistochemistry staining

Tissue sections 3 μm thick were deparaffinized, rehydrated, and subjected to antigen retrieval by pressure cooking in citrate buffer (pH 6.0). Sections were then stained with rabbit polyclonal anti-hDKK1 antibody (Cat.#3435-1, Abcam, CA, USA) and incubated with HRP-labeled anti-rabbit IgG secondary antibody. Substrate-chromogen was added, and the specimens were counterstained with hematoxylin. The intensity of DKK1 staining was evaluated using the following criteria: strong positive (scored as 3+), dark brown staining in > 50% of tumor cells completely obscuring cytoplasm; moderate positive (scored as 2+), dark brown staining in 20%∼50% of tumor cells; weak positive (scored as 1+) and no staining (scored as −), no appreciable staining in tumor cells [16]. Cases were accepted as strongly positive only if reviewers independently defined them as such.

### Statistical analysis

Descriptive statistics were conducted on all variables to evaluate normality of data. The differences in expression level of DKK1 and CA19-9 between PC patients and controls were analyzed by non-parametric Mann-Whitney U tests. Evaluation of the sensitivity, specificity, and respective areas under the curves (AUCs) with 95% of confidence interval (CI) of DKK1 and CA19-9 were analyzed by receiver operating characteristic (ROC) curve to determine cutoff line with optimal diagnostic accuracy and likelihood ratios. The correlation between serum and tissue DKK1 levels was assessed by Spearman rank correlation. Survival curves were created by the Kaplan-Meier method and survival was compared using log-rank tests. All statistical analyses were performed with SPSS software (version 18.0).
